# Host Species Adaptation of Obligate Gut Anaerobes Is Dependent on Their Environmental Survival

**DOI:** 10.3390/microorganisms10061085

**Published:** 2022-05-25

**Authors:** Daniela Karasova, Marcela Faldynova, Jitka Matiasovicova, Alena Sebkova, Magdalena Crhanova, Tereza Kubasova, Zuzana Seidlerova, Hana Prikrylova, Jiri Volf, Michal Zeman, Vladimir Babak, Helena Juricova, Jana Rajova, Lenka Vlasatikova, Petr Rysavka, Ivan Rychlik

**Affiliations:** 1 Veterinary Research Institute, 621 00 Brno, Czech Republic; karasova@vri.cz (D.K.); faldynova@vri.cz (M.F.); matiasovicova@vri.cz (J.M.); asebkova@vri.cz (A.S.); crhanova@vri.cz (M.C.); kubasova@vri.cz (T.K.); seidlerova@vri.cz (Z.S.); prikrylova@vri.cz (H.P.); volf@vri.cz (J.V.); zeman@vri.cz (M.Z.); babak@vri.cz (V.B.); juricova@vri.cz (H.J.); rajova@vri.cz (J.R.); vlasatikova@vri.cz (L.V.); 2 Department of Experimental Biology, Czech Collection of Microorganisms, Faculty of Science, Masaryk University, 625 00 Brno, Czech Republic; 3 Medi Pharma Vision Ltd., 612 00 Brno, Czech Republic; rysavka.petr@seznam.cz

**Keywords:** chicken, pig, human, penguin, gut microbiota, host adaptation, endospore, environment, spread

## Abstract

The gut microbiota of warm-blooded vertebrates consists of bacterial species belonging to two main phyla; Firmicutes and Bacteroidetes. However, does it mean that the same bacterial species are found in humans and chickens? Here we show that the ability to survive in an aerobic environment is central for host species adaptation. Known bacterial species commonly found in humans, pigs, chickens and Antarctic gentoo penguins are those capable of extended survival under aerobic conditions, i.e., either spore-forming, aerotolerant or facultatively anaerobic bacteria. Such bacteria are ubiquitously distributed in the environment, which acts as the source of infection with similar probability in humans, pigs, chickens, penguins and likely any other warm-blooded omnivorous hosts. On the other hand, gut anaerobes with no specific adaptation for survival in an aerobic environment exhibit host adaptation. This is associated with their vertical transmission from mothers to offspring and long-term colonisation after administration of a single dose. This knowledge influences the design of next-generation probiotics. The origin of aerotolerant or spore-forming probiotic strains may not be that important. On the other hand, if Bacteroidetes and other host-adapted species are used as future probiotics, host preference should be considered.

## 1. Introduction

Interactions between animal hosts and their gut microbiota considerably affect host performance and not surprisingly, composition, function, transfer and subsequent development and shaping of gut microbiota attract considerable attention. Consequently, questions on the origin of gut microbiota in newborns or the spread of gut microbiota members in human or animal communities are being addressed. Microbiota of different compositions is present in the gut of piglets before and after weaning [1]. Different neonatal microbiota is present in human infants delivered vaginally or by caesarean section [2,3]. Different microbiota colonises one-week-old chicks raised with or without contact with an adult hen [4]. Interestingly, both in humans and chickens, following initial colonisation with ubiquitously distributed *E. coli* [5,6], mothers act as important donors of Bacteroidetes but a less important source of Firmicutes, and both in humans and in chickens, this has been associated with endospore formation in Clostridia and corresponding environmental survival [7,8,9,10].

The vast majority of gut microbiota members are obligate anaerobes, which are rapidly inactivated upon exposure to air. To increase environmental survival and the likelihood of transmission, different strategies have evolved in different groups of gut microbiota [11]. The most striking adaptation is the ability of Clostridia to form endospores. Other bacteria such as *Lactobacillus* species developed aerotolerance while others, e.g., *E*. *coli*, are facultative anaerobes. Nevertheless, there is a long list of gut microbiota members, which are common to the intestinal tract but do not express any form of long-term resistance to aerobic conditions. These microbiota members belong to phyla Bacteroidetes, Verrucomicrobia (genus *Akkermansia*), Synergistetes (*Cloacibacillus*), Proteobacteria (genera *Sutterella*, *Parasuterella*, *Succinivibrio*, *Succinatimonas*, *Anaerobiospirillum*, etc.) or Firmicutes (order Selenomonadales). These represent strict anaerobes surviving under aerobic conditions for mere hours [12,13].

Accumulating evidence shows that there are biological consequences of different modes of environmental survival. Anaerobic spore-forming bacteria usually exhibit a high prevalence in their hosts which is, however, associated with low abundance [14,15,16]. If these bacteria are used for reconstitution of the gut microbiota as probiotics, then they have to be supplied continuously [17,18], which is likely to mimic their continuous environmental supply [8]. On the other hand, bacteria that do not express any specific form of extended environmental survival, e.g., *Bacteroides caecicola* or *Megamonas hypermegale* in chickens, and *Bacteroides vulgatus* or *Parabacteroides distasonis* are those which are efficiently transferred from parents to offspring [4,19]. Such bacteria, therefore, compensate for the reduced survival by mechanisms allowing them to efficiently colonise the host at the first possible occasion. Moreover, upon colonisation, these bacterial species exhibit high abundance [7,14] thus increasing their release into the environment and the likelihood of colonising the next host.

We have shown recently that different species of the genus *Bacteroides* are adapted to either chickens or humans [20]. This may indicate that bacterial species which do not efficiently survive in the environment may exhibit host species adaptation (hereafter host adaptation). If such a prediction is correct, host adaptation might be less widespread among spore-forming gut anaerobes since these can repeatedly colonise distantly related hosts from environmental sources. If this is the case, different hosts should be provided probiotics consisting of host-adapted species in which there is a decreased likelihood of colonisation from the environment. Conversely, there would be a lesser need to provide probiotic bacteria from among those bacterial species which are easily accessible in the environment.

Therefore, in this study, we compared human, pig and chicken microbiota to identify broadly distributed operational taxonomic units (OTUs) and those adapted to a single host. We show that host adaptation is characteristic for Bacteroidetes while half of the OTUs belonging to Firmicutes can be found in more than one of these hosts. These findings are of utmost practical importance. If probiotics from Firmicutes are used, there might be lower demands on their origin [21] as is the case of aerotolerant Lactobacilli [12] common in fermented dairy products but beneficial for humans. On other hand, to respect their ecology, these have to be continuously provided to cause any effect. Additionally, when selecting probiotics from phylum Bacteroidetes, their host adaptation should be considered, and a single dose administration would be likely enough to result in colonisation and a biological effect.

## 2. Materials and Methods

### 2.1. Samples

Microbiota composition in 140 samples was compared in this study. We selected human and pig samples representing omnivorous mammalian species and compared them with chickens, an avian omnivorous representative, and Antarctic gentoo penguins as another avian species of specific and different feeding preferences. Of these, 37 originated from adult hens 30–60 weeks of age (caecal contents) [22], 50 from pigs at least one month after weaning, i.e., 2 months to 3 years of age (rectal swabs) [23,24] and 44 faecal samples originated from healthy humans 20 to 55 years of age. Nine faecal droppings from gentoo penguins (*Pygoscelis papua*) were collected at the Antarctic polar station of Masaryk’s University in the coastal area of James Ross Island in 2020. Human samples were collected only from Czech citizens based on the informed consent of each person who provided the sample. Chicken and pig samples were deliberately selected from our previous studies to contain samples from different countries across the EU, thus avoiding regional bias. All these samples originated from healthy adults so as not to include samples with underdeveloped microbiota from young individuals. 

In addition to microbiota from adult individuals, we determined the caecal microbiota composition of one-week-old chicks. Specifically, 81 caecal samples were collected from control chicks from 17 different experiments presented in our recent papers [7,25], and an additional 20 caecal samples were collected from one-week-old broilers from 4 different commercial farms. These samples were not included in the comparative analysis and, instead, were used to support our hypothesis on the environmental origin of anaerobic spore-forming bacterial species in the chickens during their first week of life.

Different types of samples were compared starting from faecal samples in penguins and humans, rectal swabs in pigs and caecal contents in adult hens. Comparing samples from distal parts of the intestinal tract of humans and pigs, whether fresh faeces or rectal swabs collected during post-mortem analysis should represent very similar material. Faecal material from penguins was forced by the rules on any human activities in Antarctica which prohibit any interference or even contact with wildlife. Caecal samples collected from hen, though different from any other type of sample, is indeed the only sample of hens that, due to chicken physiology of digestion, contains strict anaerobes and is thus comparable with the samples from humans and pigs.

### 2.2. Analysis

DNA purification and sequencing of the PCR products comprising V3/V4 variable regions of 16S rRNA gene using MiSeq sequencing platform (Illumina) was performed in a single lab, by the same personnel using the same protocol and the same DNA purification kit as described previously [4,7]. Two independent calculations were performed. In the first analysis, samples originating only from humans, pigs and chickens (adult hens) were processed. In the second analysis, samples originating from humans, pigs, chickens and penguins were processed altogether. The same workflow was adopted in both analyses as follows. Quality trimming of the raw reads was performed using TrimmomaticPE v0.32 with the sliding window at 4 bp and a quality read score equal to or higher than 20 [26]. The minimal read length must have been at least 200 bp. The fastq files generated after quality trimming were uploaded into QIIME software [27], forward and reverse reads were joined, and chimeric sequences were excluded by the slayer algorithm. The resulting sequences were classified by RDP Seqmatch using the Ribosomal Database Project database with an OTU (operational taxonomic unit) discrimination level set to 97%. Principal coordinate analysis (PCoA) implemented in QIIME was used for data visualisation. Downstream analyses at the OTU level were performed only with OTUs which were present in more than half of the samples in at least one host AND such OTUs must have formed more than 0.5% of total microbiota in at least one sample. In the case of Gallus gallus, only samples from adult hens were included in the comparative analysis. Such selection was adopted to exclude rare taxa and to compare common and representative taxa for each of the hosts and the full OTU table is provided as Table S1. Multiple alignment was performed using Clustal Omega at https://www.ebi.ac.uk/Tools/msa/clustalo/ (accessed on 11 January 2022) with default settings. Obtained phylogenetic tree file was finally edited in iTol (https://itol.embl.de/ (accessed on 15 January 2022)). 

### 2.3. Presence or Absence Classification of Host-Adapted and Non-Adapted OTUs

Since microbiota composition differed among the hosts, considering abundances in samples of overall different compositions may lead to inappropriate conclusions. Next we therefore analysed the same OTU tables using only the presence or absence of each OTU in human, pig or chicken samples as the only criterion. OTUs classified as human-adapted must have been present in more human samples than in the sum of all pig and chicken samples, i.e., the difference between occurrence in human samples—(occurrence in pig + occurrence in chickens) > 0. The same was applied to define pig and chicken specific OTUs and the OTUs which always resulted in negative values in all 3 combinations, i.e., the number of occurrences in any of the two hosts was higher than in the remaining host were considered as host non-adapted.

### 2.4. Recovery of Microbiota from Wilkins Chalgren Agar after Culture under Anaerobic or Microaerophilic Conditions

The whole caecum from a donor hen 50 weeks of age from a local egg-producing farm was transferred to an anaerobic cabinet within 10 min after collection. The caecum was opened in the cabinet, caecal contents were serially diluted in pre-reduced PRAS (0.1 g magnesium sulphate heptahydrate, 0.2 g monobasic potassium phosphate, 0.2 g potassium chloride, 1.15 g dibasic sodium phosphate, 3.0 g sodium chloride, 1.0 g sodium thioglycolate, 0.5 g L-cysteine, 1000 mL distilled water; final pH 7.5 at 25 °C) and plated onto two sets of Wilkins Chalgren agar plates. One set of the plates was incubated in an anaerobic atmosphere consisting of 85% N_2_, 10% CO_2_ and 5% H_2_. The second set was transferred to incubation jars filled with Oxoid CampyGen System gaseous atmosphere for the growth of microaerophilic organisms. After two days of incubation at 37 °C, the total bacterial mass was washed from the 3rd and 4th dilution plates incubated either under anaerobic or microaerophilic conditions and the microbiota composition was determined by 16S rRNA sequencing, as described above. 

### 2.5. Data Analysis and Statistics

Zero values in the OTU table were replaced with values 1/number of reads available for each sample and a non-parametric Kruskal–Wallis test was used to identify differently abundant taxa. Differences with *p* < 0.01 were considered significant. Host adaptation was defined as follows. The abundance of chicken-specific OTU must have been significantly higher than in human or pig microbiota, irrespective of significant or insignificant difference in abundance of such OTU in human or pig microbiota. OTUs characteristic for chickens and pigs must have been significantly more abundant in chickens than in humans AND in pigs than in humans AND with an insignificant difference in abundance in chickens and pigs. OTUs with no host preference included those of insignificantly different abundance in any of the hosts but also those present in abundance order of human > chicken > pig where there was a significant difference between the two extremes, i.e., humans and pigs, but with insignificant differences between the two extremes and the middle one, i.e., human and chickens, and chickens and pigs. The same criteria were used for the definition of human or pig adapted microbiota members, or microbiota members shared by humans and chickens, or humans and pigs, as appropriate.

## 3. Results

### 3.1. Microbiota Composition in Chickens, Pigs and Humans

**Figure 1:**
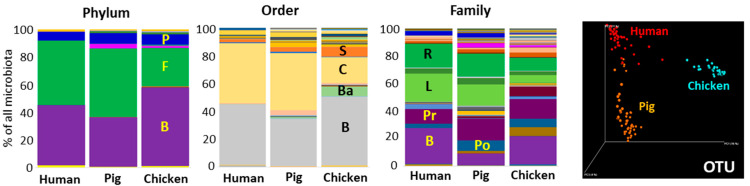
Composition of human, pig and chicken gut microbiota at selected taxonomic levels. Phylum: P—Proteobacteria, F—Firmicutes, B—Bacteroidetes. Order: S—Selenomonadales, C—Clostridiales, Ba—unclassified Bacteroidetes, B—Bacteroidales. Family: R—Ruminococcaceae, L—Lachnospiraceae, Pr—Prevotellaceae, B—Bacteroidaceae, Po—Porphyromonadaceae. For detailed composition see Table S2. OTU—weighted PCoA clustering of all human, pig and chicken samples included in this study.

Firmicutes and Bacteroidetes, with Proteobacteria as a minority phylum were recorded in the gut microbiota of humans, pigs and adult hens. Despite this, there were host-dependent differences at all taxonomic levels. The microbiota of chickens was enriched for Bacteroidetes at the expense of Firmicutes, while Firmicutes were more abundant in both mammalian species, at the expense of Bacteroidetes. The majority of Firmicutes were classified to order Clostridiales, and families Ruminococcaceae and Lachnospiraceae in humans and pigs. In chickens, Firmicutes was split between Clostridiales and Selenomonadales, the latter being represented by families Veillonellaceae and Acidaminococcaceae. Further differences were detected at the family level. Prevotellaceae and Bacteroidaceae were common in chicken microbiota. However, Prevotellaceae were of low abundance in humans and Bacteroidaceae had low representation in pig microbiota. All these differences between host species resulted in principal coordinate analysis (PCoA) clustering performed at the OTU level differentiating human, pig and chicken microbiota into separate clusters (Figure 1).

### 3.2. Specific OTUs (Non)Adapted to Different Hosts

**Figure 2:**
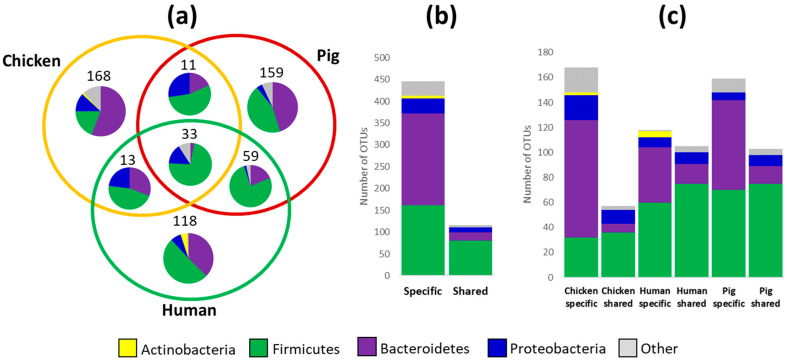
Host adaption of OTUs belonging to major phyla forming gut microbiota of humans, pigs and chickens. Panel (**a**), number of OTUs specific to or shared by different hosts. Pie charts inside Venn diagram show classification of the OTUs into 4 main phyla. Panel (**b**), host-adapted OTUs belonged mostly to Bacteroidetes while OTUs distributed in more than one host belonged mainly to Firmicutes. Panel (**c**), numbers of host-adapted and broadly distributed OTUs in each of the hosts. Similar numbers of OTUs from phylum Firmicutes were specific to a given host or present in at least one more host. On the other hand, OTUs belonging to phylum Bacteroidetes were highly specific for each of the hosts. The same colour coding applies for all 3 panels.

To compare common and representative taxa for each of the hosts, a detailed analysis at the OTU level was performed with OTUs which were present in more than half of the samples in at least one host and such OTUs must have formed more than 0.5% of total microbiota in at least one sample. Of the 561 OTUs which passed such criteria, 168, 159 and 118 OTUs represented host-adapted species associated with chickens, pigs and humans, respectively (Figure 2a). Host-adapted OTUs comprised 210 OTUs from phylum Bacteroidetes and 162 OTUs from phylum Firmicutes (Figure 2b). The remaining 116 OTUs were detected in more than one host. Of these, 81 belonged to Firmicutes and only 18 to Bacteroidetes. Within 33 OTUs, which were recorded at a similar abundance in the microbiota of all 3 hosts, only a single OTU from phylum Bacteroidetes was present while these were dominated by 23 OTUs belonging to spore-forming Firmicutes (classes Bacilli, Erysipelotrichia and Clostridia) (Figure 2a).

### 3.3. Are the Principles for Host Adaptation the Same in Each of the Hosts? 

Host adaptation was further evaluated for each host separately. In chickens, 36 OTUs belonging to phylum Firmicutes were found also in the microbiota of humans or pigs whilst 32 OTUs were chicken adapted. On the other hand, only 7 chicken OTUs belonging to phylum Bacteroidetes were found also in the microbiota of humans or pigs while 94 Bacteroidetes OTUs were chicken specific. The ratio of numbers of host-adapted to broadly distributed OTUs in Firmicutes was below a value of 1 (from 0.8 in humans to 0.93 in pigs) in all 3 hosts while the same ratio for OTUs from the phylum Bacteroidetes was 2.75, 5.14 and 13.42 for humans, pigs and chickens, respectively (Figure 2c).

### 3.4. Specific Host Adaptations

**Figure 3:**
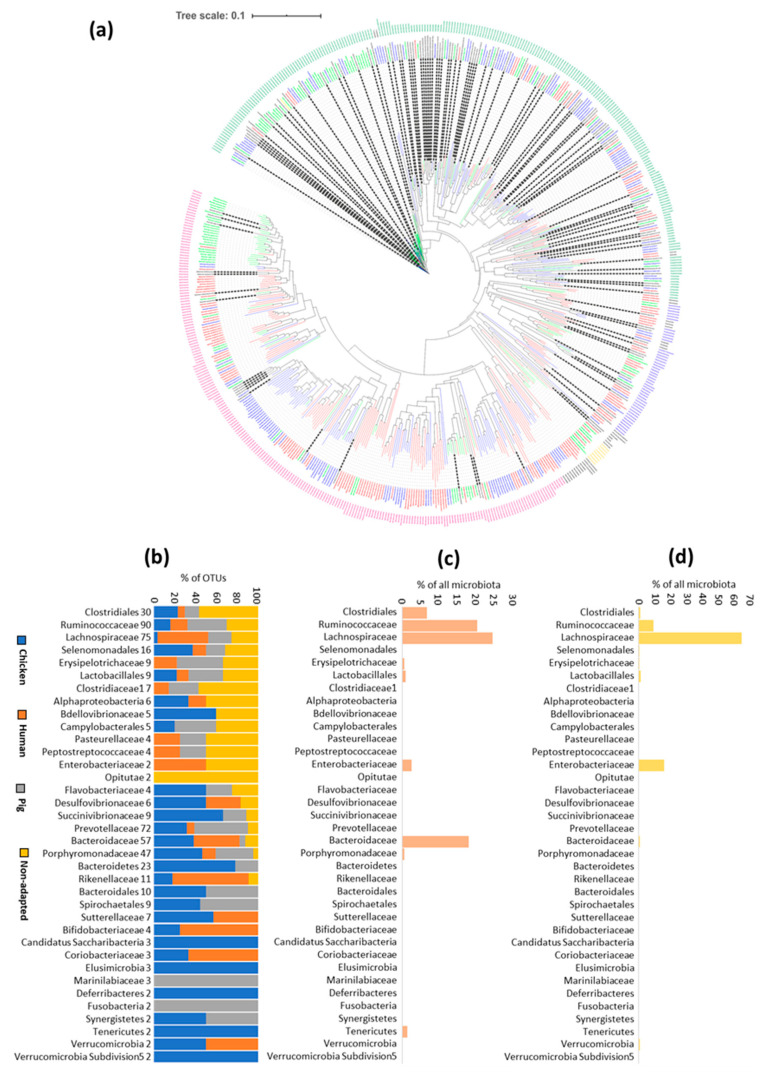
Distribution of host-adapted and broadly distributed OTUs to particular taxa. Panel (**a**), Clustal alignment of V3/V4 variable region sequences of 16S rRNA gene was used for comparison of 561 most common OTUs from chicken, human and pig microbiota. Branches in bold points towards OTUs present in two or more hosts. Colours of inner labels and branches indicate host adaptation—green indicates human-adapted OTUs, red—chicken-adapted OTUs, blue—pig-adapted OTUs, black and bold—host non-adapted OTUs. External labels indicate classification into families. Families in green belong to Firmicutes, magenta—Bacteroidetes, blue—Proteobacteria, yellow—Actinobacteria, black—all other phyla. Host non-adapted OTUs were randomly distributed mainly among Firmicutes and Proteobacteria. To zoom in, see Figure S1. Panel (**b**), numbers of OTUs belonging to particular taxa and their classification as host-adapted or non-adapted. Numbers next to taxon description show how many OTUs belonged to a given taxon. Non-adapted OTUs were common in taxa belonging to aerotolerant Proteobacteria or Firmicutes (see yellow colour e.g., in Campylobacterales, Pasteurellaceae or Lactobacillales), or anaerobic spore-forming Firmicutes (e.g., Lachnospiraceae, Ruminococcaceae, Peptostreptococcaceae or Clostridiaceae1). Panel (**c**), average abundance of the same taxa as in panel B in microbiota of seven-day-old broilers in commercial production. Panel (**d**), average abundance of the same taxa as in panel (**b**) in microbiota of seven-day-old chicks raised in contained facilities of VRI Brno. Only taxa with two or more OTUs are shown in panels (**b**,**c**).

The host-adapted or broadly distributed OTUs could be evenly distributed among different orders or families of Firmicutes or Bacteroidetes or could cluster to particular taxonomic lineages. Sequence alignment of the V3/V4 loop of 16S rRNA showed that broadly distributed OTUs belonged to families that contain species characterised by endospore formation, i.e., Lachnospiraceae, Ruminococcaceae, Erysipelotrichaceae, Clostridiaceae and Peptostreptococcaceae. Additional taxa with a high representation of broadly distributed OTUs were aerotolerant bacteria such as Lactobacillales, Campylobacterales or Enterobacteriaceae. There was one clade of *Prevotella copri*, OTUs of which were recorded both in human and porcine microbiota (Figure 3). Host-adapted taxa were recorded in all remaining taxa. Family Rikenellaceae and one clade of *Bacteroides* species were characteristic of humans. Different clades within Spirochetes, Prevotellaceae and Porphyromonadaceae were characteristic either for pigs or chickens (Figure 3). Chicken microbiota was characterised by the presence of OTUs from genera *Megamonas*, *Elusimicrobium*, *Mucispirillum* and one clade of *Bacteroides* species. There were also genera that exhibited host adaptation at the species level. Non-spore-forming *Faecalibacterium prausnitzii* was characteristic of human microbiota whilst an OTU 96.5% similar to *F*. *prausnitzii* was common in chickens. Two different OTUs within the genus *Cloacibacillus* were adapted to either chickens or pigs. Two different OTUs were found also within the genus *Akkermansia* and each of them was adapted either to chickens or humans. Genus *Phascolarctobacterium* was represented by 3 different OTUs and each of them was adapted to one of the hosts, i.e., humans, pigs and chickens (Figure 3). Neither host-adapted nor broadly distributed OTUs were therefore associated with particular taxa and instead, their characteristics corresponded with the ability to survive in an aerobic environment.

### 3.5. Microbiota of Commercially Hatched Chicks Is Enriched for Broadly Distributed Gut Anaerobes of Environmental Origin

If the species with relaxed host specificity originate from the environment, then chicks from commercial production hatched without contact with an adult hen should act as an ideal model for the identification of bacterial species of environmental origin. OTUs present in the microbiota of one-week-old broilers from commercial production belonged mainly to Lachnospiraceae, Ruminococcaceae, unclassified Clostridiales, Erysipelotrichaceae, Lactobacilli and *Escherichia* (Figure 3c). One-week-old commercial broilers were colonised also by 15 OTUs belonging to Bacteroidaceae but when their 16S rRNA sequences were BLAST compared with GenBank entries, these OTUs represented Bacteroides species which are common to humans [20]. When the same analysis was performed with the microbiota of chicks kept at contained animal houses of VRI, their microbiota was dominated by Lachnospiraceae, Ruminococcaceae and Enterobacteriaceae (*E. coli*) (Figure 3d), i.e., families comprising anaerobic spore-forming bacteria or facultative anaerobes. Lachnospiraceae, Ruminococcaceae, unclassified Clostridiales, Erysipelotrichaceae, Lactobacilli and *Escherichia* in chickens, therefore, represent microbiota of environmental origin.

### 3.6. Presence or Absence Classification of Host-Adapted and Non-Adapted OTUs 

**Figure 4:**
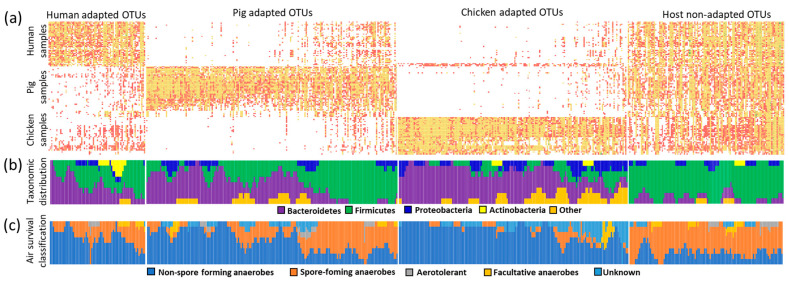
Host adaptation based on presence and absence classification. Panel (**a**), classification of OTUs as human-, pig- and chicken-adapted, or non-adapted. Each dot indicates presence of given OTU in a particular sample of human, pig or chicken origin. Heat map was used to indicate the abundance with red colour indicating abundance lower than 0.1% and yellow colour indicating abundance higher than 0.1%. Host-adapted OTUs are arranged according to the strength of their host adaptation with OTUs more to the right being present also in the non-preferred host. Panel (**b**), classification of OTUs from panel (**a**) into 4 main phyla. A sliding window of 8 OTUs was used to calculate the ratio of OTUs belonging to Bacteroidetes (magenta), Firmicutes (green), Proteobacteria (blue), Actinobacteria (yellow) and other phyla (orange). Panel (**c**), the same sliding window protocol was used but each OTU was classified as either belonging to non-spore forming (blue), spore-forming (orange), facultative anaerobe (yellow), aerotolerant (grey) and other OTUs with unknown relationship to oxygen tolerance (light blue). Classification of each OTU into spore-forming, non-spore-forming, aerotolerant or facultative anaerobe can be found in Table S3.

Next, we analysed the same dataset using the presence or absence of each OTU in human, pig or adult hen samples as the only criterion. Using such a qualitative approach, 74, 192 and 175 OTUs were classified as human, pig and chicken adapted, respectively (Figure 4a). The remaining 120 OTUs were distributed among all hosts with no preference. Within the host-adapted OTUs, some were highly adapted, e.g., present in 38 out of 44 tested human samples while this OTU was present only in 2 pig samples and a single chicken sample, and those the least adapted to humans were present in 43, 34 and 7 human, pig and chicken samples (the difference between 43 − (34 + 7) = 1, i.e., >0). When we arranged OTUs according to the extent of their host adaptation, those highly host-adapted originated from Bacteroidetes in each of the three hosts. With decreasing host preference within each host, the ratio between Bacteroidetes to Firmicutes decreased and Firmicutes dominated among the host non-adapted OTUs (Figure 4b). When the OTUs were classified according to their adaptation to air exposure, anaerobic spore formers were enriched among host-non-adapted OTUs or among the OTUs classified as host-adapted but with relaxed host preference in each host (Figure 4c).

### 3.7. Verification of Spore Formation and Host Distribution Using an Outgroup of Microbiota Composition in Antarctic Gentoo Penguins

**Figure 5:**
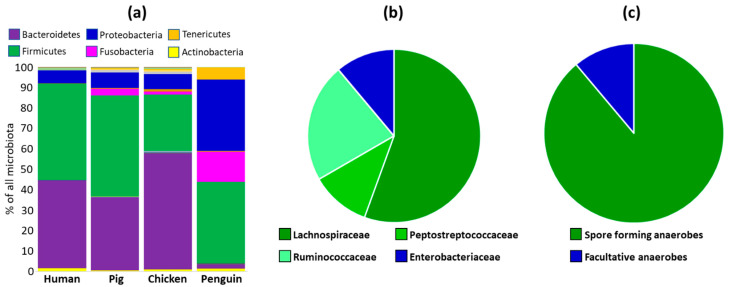
Identification of OTUs present in human, pig, chicken and gentoo penguin microbiota. Panel (**a**), faecal microbiota of wild Antarctic gentoo penguins differed from the microbiota of humans, pigs and chickens as high as the phylum level. Despite this, there were nine OTUs belonging to Lachnospiraceae (*n* = 5), Ruminococcaceae (*n* = 2), Peptostreptococcaceae (*n* = 1) and Enterobacteriaceae (*n* = 1), which were detected in more than half the samples originating from each of the hosts (Panel (**b**)). Since Ruminococcaceae, Lachnospiraceae and Peptostreptococcaceae belong to anaerobic spore forming taxa (8 OTUs in total), microbiota members shared among all four hosts were predominantly endospore formers (Panel (**c**)).

To verify that survival in an aerobic environment and spore-formation affect microbiota host adaptation, we collected outgroup samples from Antarctic gentoo penguins *Pygoscelis papua*. Faecal microbiota of penguins differed from human, pig and chicken microbiota as high as at the phylum level (Figure 5a). Therefore, we compared OTUs present in humans, pigs and chickens with penguin microbiota only using the presence or absence criterion (Table S4). The non-adapted species were defined as those present in more than half of human AND more than half of pig AND more than half of chicken AND more than half of penguin samples. There were nine OTUs fulfilling such criteria and these included *Blautia luti* (99.32% similarity over V3/V4 loop of 16S rRNA to the closest GenBank entry), *Blautia caecimuris* (99.55%), *Kineothrix alysoides* (97.5%), *Kineothrix alysoides* (95.91%), [*Ruminococcus*] *torques* (97.72%), *Terrisporobacter petrolearius* (99.09%), *Flintibacter butyricus* (97.52%), *Flavonifractor plautii* (99.77%) and *Escherichia coli* (99.78%). *E. coli* represents the facultative anaerobe and the remaining OTUs belong to anaerobic spore-forming Ruminococcaceae, Lachnospiraceae and Peptostreptococcaceae (Figure 5b,c).

### 3.8. Is the Distribution of Anaerobic Spore-Forming OTUs Indeed Dependent on Spores? 

**Figure 6:**
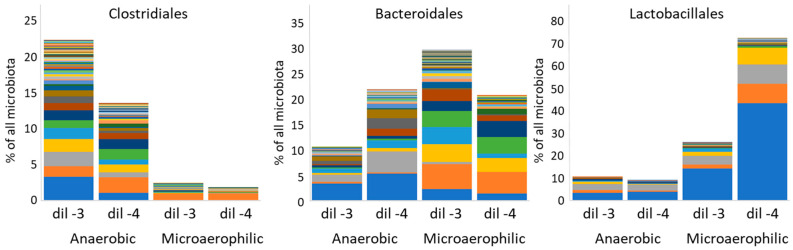
Recovery of the main gut microbiota members from Wilkins-Chalgren agar after culture under anaerobic or microaerophilic conditions. The caecal content of a donor hen was serially diluted and dilutions-3 and -4 were plated on WCHA agar plates, which were incubated under anaerobic or microaerophilic conditions. All growing colonies were washed, and the composition of microbial biomass was determined by sequencing of 16S rRNA genes. Y axis shows abundance of each order in washes from the plates with individual OTUs belonging to each order highlighted by different colours. Vegetative cells of Clostridiales grew only under strictly anaerobic conditions. Lactobacillales preferred microaerophilic conditions and Bacteroidales grew similarly both in anaerobic and microaerophilic conditions. For identification of each OTU contributing to anaerobic or microaerophilic growth of these 3 orders, please, see Table S5.

If the spread of gut spore-forming microbiota members is dependent on endospores, this might have weakened demands on the environmental survival of their vegetative cells. To test this hypothesis, serial dilutions of chicken caecal contents were plated onto Wilkins Chalgren agar and half of the plates were incubated in an anaerobic cabinet and the second half of the plates were incubated under microaerophilic conditions. Analysis of the bacterial mass washed from the plates by 16S rRNA sequencing showed that different genera of order Clostridiales grew only under strictly anaerobic conditions. Representatives of order Lactobacillales grew better under microaerophilic than strictly anaerobic conditions and representatives of order Bacteroidales, though considered strict anaerobes, formed similar biomass on agar plates incubated both under anaerobic and microaerophilic conditions (Figure 6). The vegetative cells of spore-forming Clostridiales were, therefore, more sensitive to low traces of oxygen than Bacteroidales and their spread among different hosts must be dependent on endospore formation. Bacteroidales not forming spores evolved mechanisms that allow them to survive at least temporarily under microaerophilic conditions thus increasing the time and likelihood of colonising a new host (Figure 6). This “metabolic” resistance can be further increased by bacterial clumps formed by bacteria, and biotic and abiotic substances that create a quasi-anaerobic environment [28].

## 4. Discussion

In this study we addressed a simple question; if gut microbiota of chickens, pigs and humans consist of the same major phyla Bacteroidetes and Firmicutes, and families Bacteroidaceae, Prevotellaceae, Porphyromonadaceae, Lactobacillaceae, Clostridiaceae, Ruminococcaceae and Lachnospiraceae [6,23,29,30], does it mean that their microbiota is identical or are there any host-specific differences at the OTU level? If so, are there any associations between host adaptation and taxonomic classification and biological characteristics? Results of this comparison showed that some OTUs can be found in the gut microbiota of all these species including Antarctic gentoo penguins, while others are host-adapted. Interestingly, the distribution of host-adapted and non-adapted species was not random and together with other reports allowed us to propose novel insights into the ecology of gut microbiota. The study has several limits. The number of samples from different hosts was rather moderate and though we attempted as random sample selection as possible, we could not cover all age categories evenly while age is known to affect microbiota composition. The sex of sample donors was ignored in penguins, humans and pigs while adult hen samples originated exclusively from females. If selecting shotgun sequencing, it is possible that different clones colonising different hosts will be recorded also among anaerobic spore-forming or aerotolerant gut colonisers [19]. However, we also remind that all these points should act towards increasing variability and background noise. Despite this, we recorded several less expected results.

The comparison of chicken, pig, human and penguin gut microbiota showed that bacterial species commonly found in different hosts were those capable of extended survival under aerobic conditions, i.e., either spore-forming, aerotolerant or facultatively anaerobic bacteria. The appearance of the same OTUs of spore-forming bacteria in the microbiota of a distantly related host does not mean that exactly the same clones are present in these hosts. It is likely that at the lowest taxonomic levels, different subspecies and clones will be adapted for human, pig or chicken gut [31]. Despite this, the distribution of anaerobic spore-forming bacteria among these hosts was in sharp contrast with host adaptation in bacteria not expressing any specific form of aerobic resistance. Similar conclusions on strain distribution as a function of survival in the external environment were proposed earlier [10,16,19]. Additional studies showed a low level of sharing of ethanol resistant, i.e., mostly spore-forming, OTUs between mothers and their children stressing the environmental origin of spore-forming species [8]. A significantly increased variability in the proportion of spore-forming bacteria compared with non-spore-forming bacteria was recorded earlier [15]. Since the spores mostly do not interact with an environment, these may enter many new environments, in which their vegetative cells may be subjected to allopatric speciation or clonalisation [31], thus increasing biodiversity. Finally, papers presenting spore-forming Lachnospiraceae or Ruminococcaceae in chicken gut microbiota in one or two-week-old chickens [6] in fact prove exclusively the environmental origin of these families since there is no contact between adult hens and chicks. Spore-forming Clostridiales are also enriched in the microbiota of commercial chickens compared to free-range chickens because their closed environment enables efficient reinfection with spores [22]. Anaerobic spore-forming bacterial species are therefore ubiquitously distributed in the environment from where they infect humans, pigs, chickens, penguins and likely any other warm-blooded omnivorous hosts with similar probability. Due to their broad distribution and importance in gut ecology, a new term “sporobiota” for their general description has been recently introduced [32].

Gut anaerobes with no adaptation for prolonged survival in an aerobic environment such as those from phylum Bacteroidetes, but also non-spore-forming Firmicutes from order Selenomonadales, *Sutterella*, *Parasutterella* or *Desulfovibrio* from Proteobacteria, or *Akkermansia* (Verrucomicrobia) exhibit a different profile of transmission between different hosts. *Bacteroides* are among genera that colonise the human host after faecal transplantation [33]. The specific role of non-spore-forming *Bacteroides* within the human gastrointestinal tract differing them from the broadly and highly variably distributed spore-forming bacteria was also recorded recently [14]. *Bacteroides vulgatus* but not spore-forming *Blautia wexlerae* was vertically transmitted from mothers to infants [19]. Different representatives from Bacteroidetes, Selenomonadales and anaerobic Proteobacteria are transferred from hens to chicks [4] and the same species efficiently colonise newly hatched chicks [7]. 

When all these results are combined, different ecological adaptations of gut anaerobes can be proposed. The distribution of the same microbiota species among unrelated host species, high variability within individuals of the same host species or the absence of vertical transmission, all point to the environmental origin of anaerobic spore-forming or aerotolerant gut microbiota members. The spore-dependent environmental origin also explains why their vegetative cells are more sensitive to aerobic exposure than vegetative cells of non-spore-forming species [12]. Vegetative cells of anaerobic spore-forming anaerobes may remain extremely sensitive to the aerobic environment since these bacteria use spores for transmission between two hosts. Their environmental origin means also a continuous supply of gut anaerobes. This results in a decreased need for permanent colonisation after a single dose administration and explains the low efficacy of probiotics consisting of aerotolerant Lactobacilli or Bifidobacteria [18]. The recommended continuous supply of such types of probiotics [17] is then nothing else but mimicking the natural ecology of such bacteria.

On the other hand, non-spore-forming gut anaerobes had to evolve at least a residual resistance of vegetative cells to microaerophilic conditions to increase the probability of reaching a new host [34,35]. The short time period available for their transmission also explains why such bacteria are host-adapted since transmission over greater distances to different hosts is highly reduced. This is why non-spore formers are vertically transferred both in humans and chickens. Since the likelihood of transmission of non-spore formers via an aerobic environment is reduced, non-spore-forming bacteria evolved yet not-well understood mechanisms allowing them to colonise permanently after a single inoculation [7,36]. Our results have many more consequences.

## 5. Conclusions

In this study, we tested to what extent the gut microbiota of humans, pigs and chickens are similar. We found out that the same OTUs of facultative anaerobes, aerotolerant or spore-forming bacteria can be found in the gut microbiota of humans, pigs, chickens and even Antarctic penguins. On the other hand, bacterial species not expressing any form of aerobic resistance exhibit strong host adaptation. These conclusions should be taken into consideration when selecting new types of probiotics.

## Data Availability

The raw sequence reads have been deposited in the NCBI Short Read Archive under accession number PRJNA714547. All the remaining data supporting the findings of this study are available within the article and/or Supplementary Materials.

## Supplementary Materials

The following are available online at https://www.mdpi.com/article/10.3390/microorganisms10061085/s1, Figure S1: Distribution of host-adapted and broadly distributed OTUs to particular taxa, Table S1: OTU table of all human, pig, chicken and penguin samples analysed in this study. Table S2: Composition of human, pig and chicken gut microbiota at phylum, order and family levels, Table S3: Host and environmental survival adaptation, Table S4: Human, pig, chicken and penguin host adaptation and environmental survival adaptation, Table S5: OTU table of bacterial population washed from WCHA agar incubated either under microaerophilic and strictly anaerobic conditions.
