# Supplementary material for: Host Species Adaptation of Obligate Gut Anaerobes Is Dependent on Their Environmental Survival
Source: Microorganisms. 2022 May 25;10(6):1085. doi: 10.3390/microorganisms10061085 (PMC9229247; doi:10.3390/microorganisms10061085)

Figure S1. Distribution of host adapted and broadly distributed OTUs to particular taxa. Clustal alignment of V3/V4 of 16S rRNA gene sequences was used for comparison of 561 most common OTUs from chicken, human and pig microbiota. Branches in bold points towards OTUs present in two or more hosts. Colors of inner labels and branches indicate host adaptation - green indicates human adapted OTUs, red - chicken adapted OTUs, blue - pig adapted OTUs, black - host non-adapted OTUs. External labels indicate classification into families. Families in green belong to Firmicutes, magenta - Bacteroidetes, blue - Proteobacteria, yellow - Actinobacteria, black - all other phyla. Host non-adapted OTUs were randomly distributed mainly among Firmicutes and Proteobacteria.

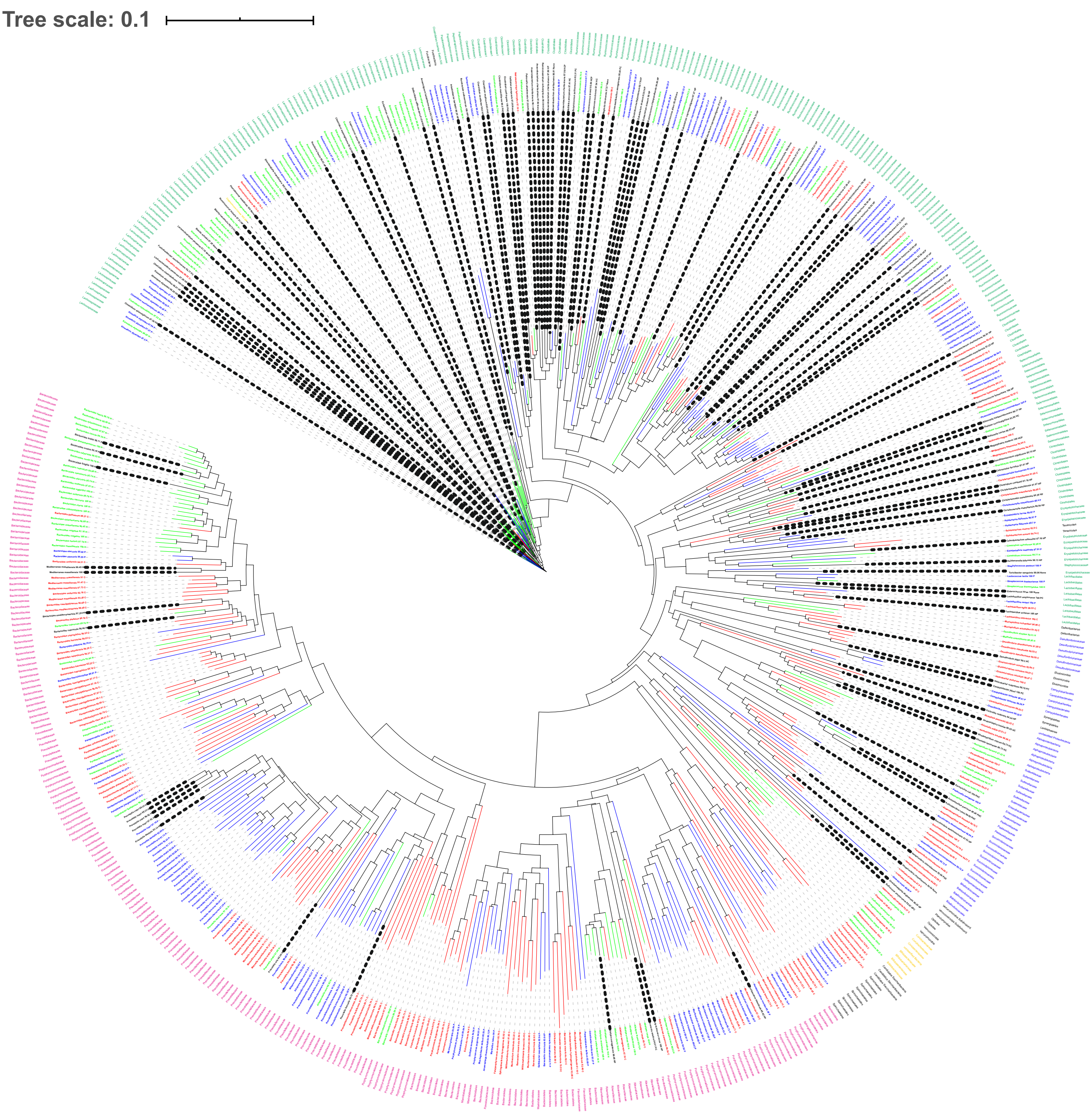

Supplement: Supplementary file 1 [file microorganisms-10-01085-s001.zip › FigureS1.pdf]
